# Citizens' national identity criteria and attitudes toward immigrants' cultural impact: ingroup–outgroup boundary setting and permeability across national contexts

**DOI:** 10.3389/fsoc.2026.1849822

**Published:** 2026-07-20

**Authors:** Melissa Lopez Reyes, Marissa Ortiz Calleja, Shayne G. Polias

**Affiliations:** Department of Psychology, De La Salle University, Manila, Philippines

**Keywords:** citizenship, civil liberties, cultural diversity, ethnoculturalism, immigrant cultures, ingroup-outgroup boundary, migrant population size, national identity

## Abstract

**Introduction:**

As international migration has increased over time, citizens in many countries have become exposed to diverse cultural ideas and practices that differ from the prevailing culture. Citizens who think that national identity is reserved for those who belong to the dominant ethnocultural group (ethnocultural view), or is achieved through a legitimation process (legitimation view), may hold less positive attitudes toward immigrants' cultural impact (I-Cultural Impact). Citizens who think that national identity is determined by respect for and affiliation with the nation (the civic view) may hold more positive attitudes. Strong civil liberties may support cultural inclusion, while a relatively large migrant population may constrain it. This paper examines the citizen-immigrant cultural nexus through the ingroup–outgroup boundary set by citizens and the broader contexts that shape boundary permeability.

**Method:**

The 34-country data analyzed are from the 2013 National Identity survey of the International Social Survey Programme (ISSP). Multilevel models with cross-level interactions were run with I-Cultural Impact as the dependent variable, national identity views as individual-level predictors, and civil liberties and migrant population size as country-level predictors.

**Results:**

Endorsers of the ethnocultural and legitimation views have more negative attitudes toward I-Cultural Impact, whereas endorsers of the civic view have more positive attitudes. Citizens in countries with strong civil liberties have more positive attitudes toward I-Cultural Impact. However, under strong civil liberties, there is a stronger association between the legitimation view and negative attitudes. This moderation effect also holds with the ethnocultural view, but only with large migrant populations. With small migrant populations and strong civil liberties, there is a weaker association between the ethnocultural view and negative attitudes.

**Discussion:**

Strong civil liberties increase the ingroup-outgroup permeability, creating tension among endorsers of the ethnocultural and legitimation views who set strict boundaries. Endorsers of the ethnocultural view perceive this tension when cultural threat becomes evident with a large migrant population. This tension persists for endorsers of the legitimation view, who rely mostly on indirect indicators of legitimation and may thus engage in gatekeeping as a matter of course. The civic view's expansive boundary-setting facilitates outgroup inclusion regardless of variations in boundary permeability.

## 1 Introduction

In many nations, citizenship has traditionally been framed around a common ethnocultural heritage (Soysal, 1996). With increasing international migration, however, peoples from diverse cultures now interact with one another as members of one nation (Held, 2007; Ozkazanc-Pan, 2019). While some multi-ethnic sectors have shown openness to diverse cultures (Nye, 2007), it can be challenging for the established population to admit immigrant cultures into the national mainstream (Cooper and Denner, 1998; Isin and Turner, 2002). Indeed, contentious discourse can arise when national identity and diverse cultural identities—while important values in themselves—are pitted against each other (Valam et al., 2006). Cultural diversity brought about by international migrants creates a need for inclusive attitudes among the nation's citizens.

This paper examines whether citizens' criteria for national identity play a role in their attitudes toward immigrants' cultural impact (**I-Cultural Impact**). Using social identity theory (Tajfel and Turner, 1986), it frames the citizens-immigrants distinction as an ingroup–outgroup boundary. As members of the ingroup, citizens set this boundary through their national identity views, or what they consider to be the essential markers of a nation's membership. These views shape their acceptance of other cultures into the national collective (Triandafyllidou, 1998). Exclusionary views correspond to a restrictive ingroup–outgroup boundary, whereas inclusionary views correspond to a more expansive boundary.

Within the ethnocultural view, what is considered essential for possessing national identity is belonging to the traditional, dominant ethnocultural group, having its ancestry, language, and religion (Herrmann et al., 2009; Jones and Smith, 2001). The legitimation view recognizes that people can rightfully claim a national identity, certainly by acquiring citizenship, but also by being born in the country or having lived there for a good part of their lives. Even though birthplace and residence are not always determinative of citizenship, these claims to national membership can be considered legitimate on moral or social grounds (Miller, 1993). In contrast, the civic view prioritizes an individual's dutiful and affective attachment to the nation. It emphasizes respect for the nation's laws and institutions, as well as self-identification with the nation's members, as essential for possessing national identity (Jones and Smith, 2001).

Boundary-setting within the ethnocultural and legitimation views is more restrictive, with strict criteria for who can be considered a member of a nation. These views exact formal requisites that immigrants may not possess or fulfill, even as they have already been engaging with the nation's civic life. In contrast, the civic view's criteria for citizenship are less rigid, expanding the boundary outward to include persons who otherwise would remain in the outgroup. As citizens come to see immigrants as part of the ingroup, they regard immigrants with greater trust and favor and, consequently, develop more positive attitudes toward their cultural influence on society. Thus, national identity views differ in the stringency with which they define the ingroup–outgroup boundary, with the ethnocultural and legitimation views being most closed to recognizing immigrants as members of the nation, and the civic view being more open.

The ingroup–outgroup boundary set by citizens' criteria for national membership is not closed but permeable, allowing movement from the outgroup to the ingroup (Armenta et al., 2017). The permeability of the citizens-immigrants boundary has been shown to be shaped by national reforms and practices (e.g., Andreouli and Howarth, 2013; Shi et al., 2017) and by demographic changes associated with migration (International Organization for Migration, 2015; Paparusso and Wihtol de Wenden, 2025).

In particular, a country's civil liberties make for a more permeable citizens-immigrants boundary because, in principle, they safeguard the freedoms and rights that protect and sustain minority cultures. When citizens encounter immigrants at this boundary, its permeability may shape citizens' perceptions of immigrants' cultures, fostering favorable perceptions through intergroup contact or less favorable ones through perceived threat (Pettigrew et al., 2010). Further, a large migrant population may compound any threat perceived by citizens, particularly those who set the boundary restrictively—especially when civil liberties uphold immigrants' cultural expression.

This paper examines the association between citizens' criteria for national membership and their attitudes toward I-Cultural Impact. It also examines whether civil liberties and migrant population size—contextual conditions that support or constrain cultural inclusion—shape citizens' attitudes toward I-Cultural Impact and, in particular, make more pronounced the tendency for citizens with restrictive criteria to view I-Cultural Impact negatively.

In this study, individual-level variables (national identity views) and country-level variables (civil liberties; international migrant population size) are analyzed across 34 countries using data from the National Identity III module of the International Social Survey Programme (ISSP Research Group, 2015). This study's objectives align with recommendations for cultural diversity research to jointly examine individual- and country-level variables (Ceobanu and Escandell, 2010) and to determine patterns of cultural inclusion associated with country contexts (Meissner and Vertovec, 2015).

## 2 National identity views as boundary-setting predictors of attitudes toward immigrants' cultural impact

Triandafyllidou (1998) regards national identity as a nation's inward-looking self-conception of “its unity, autonomy, and uniqueness” (p. 594). Within the framework of social identity theory, an analogous concept to this inward-looking conception is ingroup boundary-setting, aimed at maintaining the group's integrity and coherence. Citizens, as members of the ingroup, set this boundary by forming their own views of who belongs to the nation. The three national identity views considered in this paper are discussed in terms of whether they imply a rigid boundary with citizens' restrictive criteria for belonging to the nation, or an expansive boundary with citizens' broad criteria that enable immigrants to belong.

### 2.1 The ethnocultural view

With the ethnocultural view, the criterion for possessing national identity is belonging to the dominant ethnocultural group. With this criterion, immigrants' qualities of ancestry, religion, and language are used to mark out a nation's people. Especially in the face of increased international migration, the ethnocultural criterion can be regarded as “purist,” setting a restrictive boundary that positions immigrants, as a matter of course, outside the ingroup. Insofar as culture is largely formed within ethnic heritage and lineage, the ethnocultural view is logically linked to less favorable attitudes toward immigrants' cultures—the very criterion that endorsers of the ethnocultural view use in setting the ingroup–outgroup boundary (Ditlmann et al., 2011).

There is empirical evidence that strong endorsers of the ethnocultural view are less accepting of immigrants' cultures. Previous research shows that they tend to feel antipathy toward immigrants (Herrmann et al., 2009; Mughan and Paxton, 2006; Newman et al., 2012). Citizens' concerns about preserving a traditional, homogeneous national identity have been linked to the majority's perceived threats from those who differ from them (Ha and Jang, 2015; Huynh et al., 2015; Mughan and Paxton, 2006; Newman et al., 2012; Rose, 2007). To illustrate, strongly nationally identified Americans expressed objections toward ethnic minorities' public expression of their heritage (Yogeeswaran et al., 2014) and toward efforts to raise the status of a minority language to a lingua franca (Taras, 1998).

These findings can be understood from the perspective of social identity theory, which proposes that self-concept and self-esteem are tied to ingroup membership, such that ingroup members tend to maintain a more favorable view of their group relative to their perceived poorer qualities of the outgroup (Cooper and Denner, 1998; Herrmann and Brewer, 2004). That strong endorsers of the ethnocultural view regard immigrant cultures negatively can also be explained by social identity theory's emphasis on the ingroup's motivation to maintain a positive social identity and the attendant intergroup comparisons. Taken together, these processes underscore how the ethnocultural view operates as a restrictive boundary-setting basis for national membership.

### 2.2 The legitimation view

The legitimation view acknowledges the path to citizenship as a path to national belonging, thereby diversifying the concept of national membership by including naturalized citizens. Going beyond citizenship, the legitimation view also acknowledges social or moral rights or claims to national belonging (Dean, 2013), specifically, birthright and long-time residence. On these bases alone, without requiring formal citizenship, immigrants can claim membership in a nation regardless of whether they share the majority's ancestry and culture. In other words, the legitimation view uses rule- or rights-based rather than culturally exclusive criteria (Gibson and Hamilton, 2011).

Even as the legitimation view is a shift from ethnocultural boundary-setting, its boundary remains restrictive. While immigrants must first have a claim to national identity, they do not confer legitimacy on themselves, but it is acknowledged and then conferred on them by citizens. In other words, it is the ingroup—and not the outgroup—that defines and confirms the boundary between them.

Evidence suggests that this restrictive boundary is associated with negative attitudes toward immigrants. Instances of legitimated immigrants being marginalized or underrepresented have been documented (e.g., Bloemraad and Schönwälder, 2013). Moreover, the legitimation view may compound anti-immigrant sentiment, given that birthright and long-time residence are not necessarily equivalent to legal status. Anti-immigrant sentiments may also trigger exclusionary cultural sentiments, as suggested by findings that citizens' concern about immigrants' transgressions of law is associated with culture-based notions of national identity (Mukherjee, 2011; Mukherjee et al., 2011).

The relationship between the legitimation view and negative attitudes toward I-Cultural Impact can also be understood using Andreouli and Howarth's (2013) view of citizenship as the institutionalized way of earning social recognition. Along the path toward citizenship, migrants begin to enact a new national identity while managing their “otherness” (Andreouli and Howarth, 2013). Endorsers of the legitimation view will likewise have to manage the migrants' “otherness”. While they may recognize immigrants' legitimated national identity, they may remain reticent about having others' cultures redefine their nation.

### 2.3 The civic view

Akin to Pehrson et al.'s (2009) civic-voluntarist nationalism, the civic criteria for national identity are a person's affective identification with the nation and respect for its principles and institutions. Indeed, the civic criteria underlie shared principles that allow people from diverse cultures to share the same national identity (Malešević, 2011; Zhuojon and Hualing, 2014) and belong to a national collective (Citrin and Sides, 2008), where all assume responsibility for the nation and benefit from its conferral of rights and protection. Having the broadest criteria among the three views, the civic view sets an expansive ingroup–outgroup boundary.

Differences between the original ingroup and its new members become significantly less salient over time (Mavroudi, 2010). For example, the Shenandoah Valley in the Southwest United States, with a large White majority, has become diverse with the arrival of Hispanic labor migrants, urban migrants from Northwest Virginia, and Muslim refugees (Byrne, 2018). A theme emerging from Byrne's (2018) interviews is political and community involvement that is manifested regardless of heritage or origin.

The common goal of respect and affiliation for the nation improves intergroup relations and reduces racial prejudice (Allport, 1954; Gordon, 2017; Pettigrew and Tropp, 2006). Research shows a positive association between the civic view and positive attitudes to I-Cultural impact (Ditlmann et al., 2011; Knudsen, 1997). Persons of Chinese heritage portrayed in vignettes were judged to be more Scottish when the civic view rather than the ethnic view was experimentally prompted (Wakefield et al., 2011). Under the civic principle of the United Kingdom, the state demands that immigrants abide by the nation's laws and public values, while granting them freedom to maintain their customs and private values (Mathieu, 2017). With a civic approach to integrating immigrants into society, public perception of cultural threats from immigrants is reduced.

## 3 Civil liberties and migrant population size as permeability mechanisms that support or constrain cultural inclusion

The ingroup–outgroup boundary is not always closed, but may be permeable, allowing entry for outgroup members (Armenta et al., 2017). This study concerns the permeability of this boundary as shaped by societal factors that propagate, maintain, or reduce differences between citizens and immigrants (Plaut, 2010). Specifically, social or institutional contexts (e.g., national practices and policies) that operate at a macro level cascade down to the individual level, where they support or constrain personal viewpoints and social interactions. These macro-level influences are direct, manifesting country-level effects on individual-level factors, or they moderate, strengthening or weakening associations between individual-level factors.

Previous research has demonstrated that country-level factors moderate the relationship between individual characteristics and stance on immigration. For example, attitudes toward immigrants are informed not only by individuals' political beliefs and values but also by a country's stance on Islamic fundamentalism (Araújo et al., 2020). Similarly, cross-country analyses have shown that an open social climate reduces the negative effect of immigration-related ethnic diversity on social trust (Ziller et al., 2019).

This study examines civil liberties as an institutional framework for cultural diversity and inclusion, signaling that diverse cultural expressions belong within the national fabric. From a social identity theory perspective, this inclusive signaling corresponds to a more permeable ingroup–outgroup boundary, effectively broadening the ingroup to include the outgroup. This greater permeability, however, may resonate differently among citizens who hold inclusionary vs. exclusionary criteria for national membership.

Additionally, this study examines a large migrant population size (relative to the general population) as a demographic constraint, signaling a greater outgroup presence within the national space. From a social identity theory perspective, this increased presence reflects a more permeable ingroup–outgroup boundary, heightening threat among citizens with exclusionary criteria for national membership, particularly those already threatened by strong civil liberties.

### 3.1 Civil liberties as context: changing associations between national identity views and attitudes toward immigrants' cultural impact

Civil liberties guarantee fundamental freedoms, including freedom of expression, association, religion, and conscience, as well as rights, including the rights to privacy and equal protection under the law (The Economist Intelligence Unit, 2014; United Nations General Assembly, 1948). These freedoms and rights apply regardless of citizenship or nationality and protect individuals from discrimination (United Nations General Assembly, 1985). By safeguarding individual freedoms for all, civil liberties promote conceptions of nationhood grounded in equality rather than cultural or religious affiliation (Helbling et al., 2016; Zhuojon and Hualing, 2014). This rights-based framework protects and promotes cultural rights (Held, 2007; Kunovich, 2009) and shifts the focus from immigrants' assimilation to a country's multicultural view (Kymlicka, 1995). As a result, individuals and groups can freely and openly live and express their cultural identities, and cultural diversity becomes woven into the fabric of national life.

Thus, strong institutional assertions of civil liberties signal cultural openness and inclusivity, encouraging citizens to view migrants as co-equal members of society with the same rights and freedoms in civic and cultural life. In contrast, societies where rights are not considered natural or absolute and where civil liberties are inconsistently applied to immigrants signal resistance to cultural inclusivity (Cooper and Denner, 1998; Pakulski, 1997). From the lens of social identity theory, this signaling of inclusivity makes citizens-immigrants boundary more permeable: citizens become more open to contact with immigrants, facilitating social integration. As a result, individuals and groups that would otherwise be excluded from the ingroup are incorporated into it.

Notably, the greater boundary permeability afforded by civil liberties may resonate with citizens who hold an inclusionary view of national identity (civic view). This is supported by the finding that in countries with inclusive policies and where citizens have homogeneous civic conceptions of nationhood, citizens are more open to diverse worldviews (Sarrasin et al., 2020). Conversely, this same boundary permeability may pose a threat to citizens who hold exclusionary views (ethnocultural and legitimation views). Apart from questioning the cultural rights afforded to immigrants, citizens with exclusionary views may perceive strong civil liberties as extending protections and recognitions to the outgroup whose claims to national membership they regard as less legitimate than those of the established ingroup. In other words, while strong civil liberties—with their greater boundary permeability—strengthen the positive association between the civic view and attitudes toward I-Cultural Impact, they also strengthen the negative association with the ethnocultural and legitimation views.

Indeed, resistance to institutional support of multiculturalism has been documented. When the minority poses a threat to the majority, the majority seeks to reaffirm its national identity, often through nativist arguments. Japanese adherents of cultural homogeneity have been shown to support multiculturalism in principle but resist extending to ethnic minorities the same rights they enjoy (Nagayoshi, 2011). When invoking a single national identity within a multicultural context, people would position the majority culture as the embodiment of national identity. Swiss survey respondents with moderate anti-immigrant attitudes regard as a threat immigrants who fail to adapt to Swiss culture, but show reduced opposition to immigrants who adapt (Falomir-Pichastor and Mugny, 2013).

### 3.2 Migrant population size as demographic constraint: changing associations between national identity views and attitudes toward immigrants' cultural impact across civil liberties contexts

This study examines migrant population size as a demographic constraint that, when large relative to the general population, increases citizens' exposure to immigrants and renders the citizens-immigrants boundary more permeable. This increased permeability, in turn, poses a threat to citizens with exclusionary views, especially in contexts of strong civil liberties.

A larger migrant population is more visible in everyday contexts, accentuating ethnocultural diversity and migrants' growing cultural influence. From a social identity theory perspective, this scenario makes the citizens-immigrants boundary more permeable. Citizens respond to this increased permeability depending on their national identity view. Those who hold the inclusionary civic view are inclined to admit immigrants to the ingroup, effectively opening themselves to cultural diversity. Citizens who hold the ethnocultural view may seek to maintain a clear ingroup–outgroup boundary to prevent dilution of the ingroup's cultural homogeneity (Craig et al., 2017; Ha and Jang, 2015; Quillian, 1995). Citizens who subscribe to the legitimation view may perceive immigrants as leveraging their legitimated status to gain a stronger position (Wagner et al., 2006) and even question whether the new ideas and cultures the immigrants bring are good for the country (Simon and Sikich, 2007).

Additionally, this research examines whether the diversity-constraining influence of civil liberties on citizens with exclusionary national identity views becomes more pronounced in countries with large migrant populations. When migrant populations are small, the ingroup–outgroup boundary remains relatively closed and citizens with exclusionary views experience little additional threat. A large migrant population, however, increases boundary permeability by expanding the social space shared by citizens and immigrants. Citizens may then perceive that “interacting (with immigrants) is often difficult due to differences in cultural values and languages” (Stephan et al., 2005, p. 15). Greater boundary permeability may also reinforce any perceptions within the ingroup that immigrants are gaining cultural recognition and social influence within the national community. As a result, citizens with exclusionary views may perceive a greater threat to the ingroup's social order, beyond any threat that they may already experience under conditions of strong civil liberties. Thus, a large migrant population is expected to strengthen the diversity-constraining influence of civil liberties on the relationship between exclusionary national identity views and unfavorable attitudes toward immigrants' cultural impact.

## 4 Theoretical framework and hypotheses

**Figure 1:**
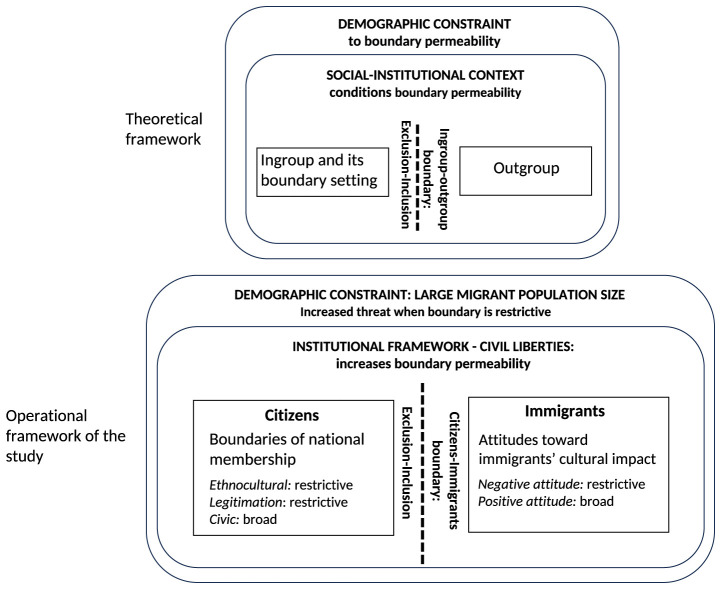
Theoretical and operational frameworks of the study.

The frameworks used in this study are shown in Figure 1. The theoretical framework (top panel) proposes that the boundary between ingroup and outgroup is set by ingroup members and varies in restrictiveness. Restrictive boundaries admit fewer members into the ingroup, whereas broader boundaries admit more, including those that other ingroup members may recognize as not belonging. It is at this boundary where ingroup and outgroup members encounter each other and where members' attitudes toward the outgroup are formed. The boundary is not necessarily closed but possesses varying degrees of permeability, or the ease of movement of outgroup members into the ingroup. An impermeable boundary restricts such movement, whereas a permeable boundary allows outgroup members to join the ingroup. Boundary permeability is conditioned by the social-institutional context and may be further constrained by a country's demographic composition.

The operational framework (bottom panel) translates these theoretical concepts into the study's variables. Individual citizens of the ingroup set the boundary by their views of who can rightfully belong to the nation. Exclusionary views of national identity (ethnocultural and legitimation) set a restrictive boundary, and the inclusionary view (civic) sets a broad boundary. The institutional framework of civil liberties increases boundary permeability by signaling inclusion and equal rights. Such signaling is seconded by citizens who hold the inclusionary view. Citizens with exclusionary views, however, may perceive greater permeability as a cultural threat, leading to exclusionary attitudes toward immigrants. Moreover, this exclusionary response is expected to become more pronounced under the demographic constraint of a large migrant population that further increases the salience of the group boundary, thereby increasing perceived threat.

Accordingly, this study examines citizens' attitudes toward immigrants' cultural impact through the lens of boundary setting and boundary permeability—the processes through which citizens and immigrants encounter one another's lives, values, and cultures. In light of this framework and the supporting arguments and literature discussed above, the following hypotheses are proposed:

*H1: The ethnocultural and legitimation views are associated with more negative attitudes toward immigrants' cultural impact, whereas the civic view is associated with more positive attitudes*.
*H2: Citizens tend to hold more positive attitudes toward immigrants' cultural impact in countries with stronger civil liberties*.
*H3: In countries with stronger civil liberties, (a) the associations of the ethnocultural and legitimation views with negative attitudes toward immigrants' cultural impact are stronger, and (b) the association of the civic view with positive attitudes toward immigrants' cultural impact is stronger*.
*H4: The moderating effect of civil liberties described in H3(a) is conditional on migrant population size. With larger migrant populations, stronger civil liberties are associated with stronger relationships between the ethnocultural and legitimation views and negative attitudes toward immigrants' cultural impact. With smaller migrant populations, stronger civil liberties are associated with weaker relationships between the ethnocultural and legitimation views and negative attitudes*.

## 5 Method

The data analyzed were from the 2013 National Identity III module of the International Social Survey Programme (ISSP; Ganzeboom, 2017; ISSP Research Group, 2015). The following details in this section are from ISSP's study monitoring document (Joye and Sapin, 2016).

### 5.1 Sampling procedure

The 34 countries surveyed are in Europe (25), East Asia and the Pacific (4), and there is one country each from Africa, Latin America, the Middle East, South Asia, and North America. The countries and sample sizes are listed in Section 1 of the Supplementary material.

Sampling procedures differed across countries, with some using simple random sampling and others multistage stratified random sampling. Countries differed in the stratification variables used (e.g., population register, electoral roll, telephone directory). The number of stages varied across countries where multistage sampling was done. Frames in multistage sampling consisted of either addresses, households, named individuals, or areas.

ISSP documentation reported no common weighting scheme across countries that is usable for cross-country comparisons (GESIS—Leibniz Institute for the Social Sciences, 2015b). The absence of sampling weights does not appear to have affected statistical inference, however. The coefficient of variation of 0.30 suggests no strong imbalance in country sample sizes, reducing the likelihood that a few countries disproportionately influence the estimates. The use of probability-based sampling by all countries strengthens cross-country comparability. The mostly comparable robust and conventional standard errors indicate no effects of the absence of weights on the variance estimation.

### 5.2 Participants

Citizens' data were used in the analysis. Of the 46,935 respondents, 44,906 (96%) are citizens. Data from respondents who did not specify their sex, age, or parents' citizenship (analyzed as covariates) were excluded from the analyses (*n* = 352; <1% of citizens), resulting in a sample of 44,554.

ISSP used a minimum age cut-off of 18 years, except in 5 countries with lower cut-offs (no lower than 15 years). There was no maximum age cut-off, except for 5 countries (with cut-offs of 74, 79, 80 years).

Respondents' mean age is 47.66 (*SD* = 17.51), 53% of the respondents are female, 45% live in urban areas, 40% have completed at least postsecondary education, and 93% indicated that both their parents are citizens of their country. The respondents' demographic profiles by country are shown in Section 1 of the Supplementary material.

### 5.3 Measures

#### 5.3.1 National identity views and attitude toward immigrants' cultural impact

Data were obtained from the 2013 National Identity III survey (Ganzeboom, 2017; ISSP Research Group, 2015). Shown in Table 1 are the questions and response options for the ethnocultural view (**V-Ethnocultural**), the legitimation view (**V-Legitimation**), the civic view (**V-Civic**), and attitude toward I-Cultural Impact. For each variable, factor and reliability analyses were conducted on the entire sample and individual countries. Shown in Table 2 are the proportion of total variance explained, the average item loading on the factor, Cronbach's α, and average inter-item correlation for the entire sample and the minimum and maximum across countries. Country results are reported in Section 2 of the Supplementary material. Factor analysis with the principal components extraction method confirmed the one-factor structure of the variable. While modest, the reliability values are standard for scales that have a small number of items (e.g., Simms and Watson, 2007; Vauclair et al., 2015).

**Table 1:** Items for national identity views and attitude toward immigrants' cultural impact from the National Identity III module of the International Social Survey Programme.

Question, Likert-scale response options, and scoring	Item
National identity views^a^	
*Some people say that the following things are important for being truly [nationality; e.g., Belgian]. Others say they are not important. How important do you think each of the following is…* 4: very important to 1: not important at all ^a^	**V-Ethnocultural** *to have [nationality] ancestry to be able to speak [dominant language's] to be a [dominant religion]*
	**V-Legitimation** *to have been born in [country] to have [nationality] citizenship to have lived in [country] for most of one's life*
	**V-Civic** *to respect [nationality] political institutions and laws to feel [nationality]*
**Attitude toward immigrants' cultural impact**	
*There are different opinions about immigrants from other countries living in [country]. (By “immigrants” we mean people who come to settle in [country]. How much do you agree or disagree with…* 5: agree strongly to 1: disagree strongly	*Immigrants improve [country's nationality] society by bringing new ideas and cultures [Country's] culture is generally undermined by immigrants* (reversed-scoring)

Quoted from (GESIS—Leibniz Institute for the Social Sciences 2015a,b).

^a^The scales for South Africa and the Netherlands included a neutral option and these scales were transposed to the other countries' scales.

**Table 2:** Factor and item reliability analyses for national identity views and attitude toward I-Cultural Impact.

Variable	V-Ethnocultural (3 items)	V-Legitimation (3 items)	V-Civic (2 items)	Attitude toward I-Cultural Impact (2 items)
Proportion of total variance explained by a one-factor solution				
Entire sample	0.56	0.69	0.67	0.66
Minimum across countries	0.46	0.50	0.56	0.51
Maximum across countries	0.69	0.79	0.78	0.85
Average item loading on the factor				
Entire sample	0.73	0.83	0.82	0.82
Minimum across countries	0.67	0.70	0.75	0.72^a^
Maximum across countries	0.83	0.89	0.88	0.90
Internal consistency reliability (Cronbach's α)				
Entire sample	0.60	0.77	0.51	0.49
Minimum across countries	0.41	0.48	0.21	0.11^b^
Maximum across countries	0.77	0.87	0.72	0.76
Average inter-item correlation				
Entire sample	0.33	0.53	0.34	0.32
Minimum across countries	0.18	0.25	0.13	0.06^c^
Maximum across countries	0.54	0.69	0.56	0.61

^a^Does not include the outlying value of 0 for both India and Mexico. ^b^Does not include the outlying values for India (−1.02), Mexico (−0.04), the Philippines (0.02), and Turkey (0.01). ^c^Does not include the outlying values for India (−0.33), Mexico (−0.02), the Philippines (0.01), and Turkey (0.00).

#### 5.3.2 Migrant population size relative to the general population

The percentage of a country's population composed of international migrants (**Migrant-%-Population**) was computed using 2013 country-level data on international migrant stock and population size (United Nations Department of Economic and Social Affairs Population Division, 2013). The statistics for Taiwan on foreign residents and population size were obtained, respectively, from Taiwan's Ministry of the Interior National Immigration Agency Republic of China (2013) and NationMaster (2013).

#### 5.3.3 Civil liberties

The civil liberties component of the Democracy Index 2013 (The Economist Intelligence Unit, 2014) was used. It was measured on a 0-to-10 scale and derived from experts' assessments, with some items from the World Values Survey. Indicators include free media with robust and diverse coverage; freedom of religion, expression, protest; citizens' equal treatment under the law, basic security, personal freedoms, rights to property and business, opportunity to petition government about grievances; and, public perception on human rights protection, no significant discrimination due to race, color, or creed, and non-invocation of threats to curb civil liberties.

The country means for the individual-level variables (national identity views and attitude toward I-Cultural Impact) and the country statistics on Migrant-%-Population and civil liberties are in Table 3.

**Table 3:** Country statistics on migrant-%-population and civil liberties and country means for national identity views and attitude toward I-Cultural Impact.

Country	Migrant-%-Population	Civil liberties	Mean attitude I-Cultural Impact	Mean V-Ethnocultural	Mean V-Legitimation	Mean V-Civic
Belgium	10.4	9.41	2.83	2.56	3.17	3.38
Croatia	17.6	8.24	3.18	2.94	2.92	3.11
Czech Republic	4.0	9.41	2.81	2.88	3.34	3.29
Denmark	9.9	9.41	3.44	2.65	2.98	3.51
Estonia	16.3	8.82	3.02	2.53	2.87	3.48
Finland	5.4	9.71	3.28	2.47	2.90	3.37
France	11.6	8.53	3.01	2.61	3.11	3.70
Georgia	4.4	5.88	2.72	3.54	3.23	3.61
Germany	11.9	9.12	3.38	2.63	2.97	3.25
Great Britain	12.4	9.41	2.91	2.83	3.27	3.37
Hungary	4.7	8.24	3.09	3.12	3.27	3.36
Iceland	10.4	9.71	3.77	2.70	3.00	3.44
India	0.4	9.41	2.92	2.98	3.44	3.40
Ireland	15.9	10.00	3.51	2.44	3.30	3.16
Israel	26.5	5.59	2.79	2.80	2.99	3.31
Japan	1.9	9.41	3.28	2.63	3.13	3.15
Latvia	13.8	9.12	3.01	2.71	3.17	3.37
Lithuania	4.9	9.71	3.13	3.03	3.09	3.13
Mexico	0.9	7.35	3.03	3.03	3.29	3.25
Netherlands	11.7	9.41	3.37	2.60	3.05	3.38
Norway	13.8	10.00	3.25	2.68	3.12	3.60
Philippines	0.2	9.12	3.16	3.67	3.72	3.64
Portugal	8.4	9.41	3.46	2.83	3.15	3.34
Russia	7.7	4.12	2.58	3.26	3.39	3.40
Slovakia	2.7	9.12	2.95	3.07	3.29	3.21
Slovenia	11.3	8.82	3.14	2.56	2.86	3.20
South Africa	4.5	8.53	2.80	3.43	3.65	3.48
South Korea	2.5	8.53	3.19	2.95	3.25	3.30
Spain	13.8	9.41	3.31	2.82	3.15	3.14
Sweden	15.9	10.00	3.44	2.29	2.69	3.44
Switzerland	28.9	9.41	3.52	2.70	2.99	3.40
Taiwan	2.2	9.41	3.37	2.42	3.02	3.49
Turkey	2.5	3.82	2.60	3.43	3.39	3.34
United States	14.3	8.53	3.53	2.84	3.22	3.47

### 5.4 Procedure

Data-gathering duration varied across countries from 2 weeks to 3 months. The earliest start date was October 2012; the latest end date was March 2015. Modes of interviews differed across countries, with some countries using one mode and others using multiple modes. The modes of interviews were face-to-face paper-and-pencil interview, computer-assisted interview, self-administered questionnaire, or interactive self-administered questionnaire (GESIS—Leibniz Institute for the Social Sciences, 2015a).

## 6 Results

### 6.1 Multilevel modeling procedures

Missing data on the items for V-Ethnocultural, V-Legitimation, and V-Civic, and attitude toward I-Cultural Impact averaged 2.37%, 1.68%, 2.16%, and 5.01%, respectively. To impute values on missing data, a neural network was trained to learn data patterns from 35,000 randomly selected records with complete data (the training data). The remaining complete records were used as test data. Training iterations were terminated when training prediction accuracy continued to improve without a corresponding increase in test prediction accuracy. (See Section 3 of the Supplementary material).

The level-1 predictors are V-Ethnocultural, V-Legitimation, and V-Civic; the level-1 covariates are “neither parent a citizen” (coded 1; otherwise, 0), sex (female coded 0; male coded 1), and education (0–6 scale). The correlation matrix for level-1 variables is shown in Table 4. The level-2 predictors are Migrant-%-Population and civil liberties, with a correlation of 0.07 (ns).

**Table 4:** Correlations among individual-level variables.

Variable number	Variable	*M*	*SD*	1	2	3	4	5	6
1	Sex^a^	0.47	0.50	–					
2	Education^b^	3.27	1.59	0.00	–				
3	Neither parent a citizen^c^	0.07	0.26	0.00	0.04^*^	–			
4	V-Ethnocultural	2.86	0.76	−0.04^*^	−0.23^*^	−0.11^*^	–		
5	V-Legitimation	3.18	0.73	−0.01	−0.21^*^	−0.10^*^	0.64^*^	–	
6	V-Civic	3.38	0.64	−0.02^**^	−0.01	−0.01^***^	0.40^*^	0.40^*^	–
7	Attitude toward I-Cultural Impact	3.11	0.91	−0.02^**^	0.23^*^	0.08^*^	−0.31^*^	−0.27^*^	−0.07^*^

^a^Female: 1, male: 0. ^b^Highest completed education: no formal education: 0, primary school: 1, lower secondary (does not allow entry to university): 2, upper secondary (allows entry to university): 3, post-secondary, non-tertiary: 4, lower level tertiary, first stage: 5, upper level tertiary: 6. ^c^Neither parent a citizen: 1, at least one parent a citizen: 0.

^*^p < 0.001. ^**^p < 0.01. ^***^p < 0.05.

In the multilevel models conducted, V-Ethnocultural, V-Legitimation, V-Civic, and education were centered around the country mean. Migrant-%-Population and civil liberties were standardized (i.e., *z*-scores). The interaction term Migrant-%-Population X Civil Liberties represents the standardized product of these two *z*-scores. While this additional scaling transforms the metric of the coefficients of terms that include this interaction, the interpretation of these coefficients is confined to the sign (direction) and statistical significance, not including the magnitude. Further, this linear scaling does not alter the *t*-statistics and *p*-values.

A series of nested multilevel models was run (West et al., 2011) using HLM 7.03 (Raudenbush et al., 2013). Model 0 (the baseline model) is a fully unconditional, random-intercepts model with attitude toward I-Cultural Impact as the level-1 outcome variable, which is modeled as a sum of a country-specific intercept and a random residual. Level-1 predictors and covariates were added in Model 1; level-2 predictors in Model 2; two-way cross-level interaction terms in Model 3; and three-way cross-level interaction terms in Model 4. Shown in Section 4 of the Supplementary material are the level-1 and level-2 model specifications of the intercepts, level-1 and level-2 residuals, and regression coefficients, including which are fixed and random effects.

The restricted maximum likelihood method of estimation was used to ensure unbiased estimates of variance components and of the intraclass correlation coefficient ICC given the relatively small number of countries (level-2 units) (McNeish, 2017).

The estimates of the within-country and between-country variances are 0.7363 and 0.0843, respectively. The intraclass correlation coefficient *ICC* is 0.1027, indicating that 10.27% of the variance in attitude toward I-Cultural Impact is accounted for by country-level differences. This *ICC* value is comparable to those of baseline multilevel models from various ISSP surveys (Staerklé et al., 2010: *ICC* = 0.0621, 0.0758, 0.1006; van der Meer et al., 2009: *ICC* = 0.05, 0.09, 0.14; and Van de Walle et al., 2015: *ICC* = 0.1370, 0.1062).

The within-country variances at Model 0 and Model 1, respectively, are 0.7363 and 0.6533, resulting in 11.27% reduction, which is significant based on the approximate likelihood ratio test, χ^2^(6) = 6,172.96, *p* < 0.0001. The between-country variances at Model 1 and Model 2, respectively, are 0.0855 and 0.0461, resulting in 46.08% reduction, which is significant, χ^2^(2) = 12.76, *p* < 0.01.

### 6.2 Multilevel modeling diagnostics

The data diagnostics for Model 4, described below, did not reveal sizable deviations from the assumptions of homogeneity of variances for level-1 residuals or from the normality of level-1 and level-2 residuals.

As regards the normality of level-1 and level-2 residuals, the normal Q–Q plots of level-1 residuals per country and pooled across countries are approximately linear, suggesting that the level-1 residuals are normally distributed. The plot of mdist (Mahalanobis distance) vs. chipct (their expected values) neatly resembles a 45-degree line, suggesting that the level-2 residuals are normally distributed.

As regards the homogeneity-of-variance assumption for level-1 residuals, although *c*^2^ for homogeneity of variance is significant and indicates differences in within-country variances (*c*^2^ = 1,049.42, df = 33, *p* < 0.0001), the box-and-whisker plots of level-1 residuals for the 34 countries show similar profiles. The ratio of the largest to smallest within-country variance of level-1 residuals is 1.68.

With these results of the data diagnostics, it is reasonable to treat the outcome variable on an approximately interval or interval level of measurement, as was done, too, in other studies that used Likert-type scales with one or a few items (Ariely, 2018; Sumino, 2014; Tam and Chan, 2018).

### 6.3 Sensitivity analyses

Sensitivity analyses were conducted to assess the robustness of the model's fixed effects across nested models, alternative model specifications, and country exclusions.

Across the nested models, the regression coefficients are stable in direction, magnitude, and statistical significance. Individual-level main effects remain stable with the addition of country-level predictors and two- and three-way interaction terms. Country-level main effects remain stable with the addition of two-and three-way interaction terms. Two-way interaction effects remain stable with the addition of three-way interactions.

Likewise, regression coefficients remain stable across alternative model specifications, which include the inclusion of country-level moderators external to the models reported in this paper, models with robust vs. conventional standard errors, and different centering and standardization procedures. Further, models specifying random variations in individual-level coefficients were run, yielding high reliability estimates for the ethnocultural and legitimation slopes, thus, supporting the inclusion of cross-level interaction effects. Variance-covariance matrices show some interdependence among higher-order interaction effects but no unusually large variances. Model variations with or without two countries with poor factor and reliability statistics, including negative Cronbach's αs (reversed-scoring was double-checked), show stable regression coefficients.

The exceptions are the regression coefficients for two cross-level interactions, namely, Migrant-%-Population X Civic View; and Migrant-%-Population X Civil Liberties X Legitimation, each interaction associated with a hypothesis. These effects are not robust, in various model specifications, to the removal of the two countries with poor factor and reliability statistics.

Overall, sensitivity analyses indicate robustness of the empirical findings against statistical and measurement artifacts. Accordingly, the results and discussion below are limited to stable, significant effects.

### 6.4 Hypothesis-testing with the multilevel models

This section reports the results of the main and interaction effects corresponding to this study's hypotheses. The estimates and statistical significance of the added fixed effects in Models 1 through 4 are shown in Table 5. The tests of main effects for level-1 predictors are based on Model 1, and those for level-2 predictors on Model 2. The tests of two-way interaction effects are based on Model 3, and of three-way interaction effects on Model 4.

**Table 5:** Fixed-effects estimates for the nested multilevel models tested in mixed-order form.

Effects	Model 0		Model 1		Model 2		Model 3		Model 4	
			Level-1 predictors and covariates added		Level-2 predictors added		Two-way cross-level interactions added		Three-way cross-level interactions added	
	Coeff	*SE*	Coeff	*SE*	Coeff	*SE*	Coeff	*SE*	Coeff	*SE*
Intercept and covariates										
Intercept	3.14^*^	0.05	3.16^*^	0.05	3.16^*^	0.04	3.16^*^	0.04	3.16^*^	0.04
Neither parent is a citizen^a^			0.27^*^	0.02	0.27^*^	0.02	0.26^*^	0.02	0.25^*^	0.02
Sex^a^			−0.05^*^	0.01	−0.05^*^	0.01	−0.05^*^	0.01	−0.05^*^	0.01
Education^a^			0.08^*^	0.01	0.08^*^	0.01	0.08^*^	0.01	0.08^*^	0.01
H1: level-1 predictors										
V-Ethnocultural			−0.25^*^	0.01	−0.25^*^	0.01	−0.25^*^	0.01	−0.26^*^	0.01
V-Legitimation			−0.16^*^	0.01	−0.16^*^	0.01	−0.16^*^	0.01	−0.16^*^	0.01
V-Civic			0.12^*^	0.01	0.12^*^	0.01	0.11^*^	0.01	0.11^*^	0.01
H2: level-2 predictors										
Migrant-%-Population					0.06	0.04	0.06	0.04	0.06	0.04
Civil Liberties					0.19^*^	0.04	0.19^*^	0.04	0.19^*^	0.04
H3: two-way cross-level interactions involving migrant-%										
Migrant-%-Population X V-Ethnocultural							−0.04^*^	0.01	−0.06^*^	0.01
Migrant-%-Population X V-Legitimation							−0.03^*^	0.01	−0.03^*^	0.01
Migrant-% Population X V-Civic							−0.03^*b^	0.01	−0.03^*^	0.01
H3: two-way cross-level interactions involving civil liberties										
Civil Liberties X V-Ethnocultural							−0.01	0.01	−0.01	0.01
Civil Liberties X V-Legitimation							−0.07^*^	0.01	−0.07^*^	0.01
Civil Liberties X V-Civic							0.01	0.01	0.01	0.01
H4: three-way cross-level interactions										
Migrant-% X Civil Liberties X V-Ethnocultural									−0.03^*^	0.01
Migrant-% X Civil Liberties X V-Legitimation									−0.02^**b, c^	0.01
Migrant-% X Civil Liberties X V-Civic									0.01	0.01

^a^Coded as indicated in Table 4. ^b^In various model specifications, not robust to the removal of countries with poor factor and reliability statistics. ^c^The two-way Civil Liberties X V-Legitimation interaction plots are similar across small, median, and large Migrant-%-Population.

Model 0: *df* = 33 for the intercept. Model 1: *df* = 33 for the intercept, *df* = 44,514. Model 2: *df* = 31 for the intercept, Migrant-%-Population, and civil liberties, *df* = 44,514 for the rest. Model 3: *df* = 31 for the intercept, Migrant-%-Population, and civil liberties, *df* = 44,508 for the rest. Model 4: *df* = 31 for the intercept, Migrant-%-Population, and civil liberties, *df* = 44,505 for the rest.

^*^p < 0.001. ^**^p < 0.01.

#### 6.4.1 Attitude toward immigrants' cultural impact predicted by national identity views and country context

Consistent with *H1*, citizens who subscribe more to V-Ethnocultural and V-Legitimation have significantly more negative attitudes toward I-Cultural Impact, whereas citizens who subscribe more to V-Civic have more positive attitudes.

*H2* is supported. Civil liberties are significantly associated with positive attitudes toward I-Cultural Impact.

#### 6.4.2 Moderation by migrant population size

*H3*, particularly pertaining to the moderating effects of Migrant-%-Population, is partially supported. Tests of two-way interactions indicate that Migrant-%-Population moderates the effects of V-Ethnocultural and V-Legitimation. The Migrant-%-Population X V-Civic interaction effect is not robust, in various model specifications, to the removal of countries with poor factor and reliability statistics.

**Figure 2:**
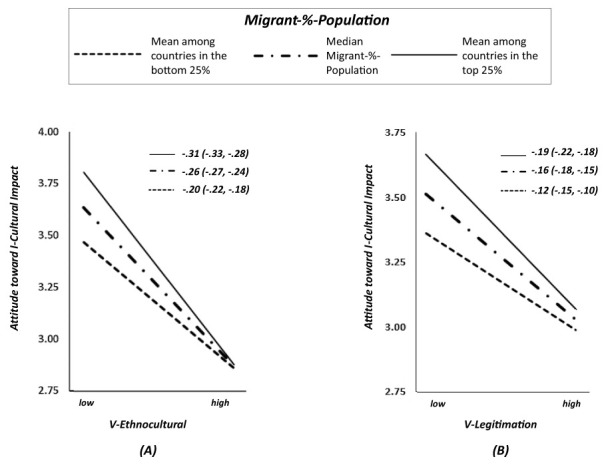
Migrant-%-Population moderates the effects of V-Ethnocultural and V-Legitimation on attitude toward I-Cultural Impact.

Preacher's online calculation facilities for hierarchical linear modeling (Preacher et al., 2006; Preacher, 2010) were used to probe the significant moderation effects. Figure 2 presents interaction plots for V-Ethnocultural and V-Legitimation predicting attitude toward I-Cultural Impact at low, median, and high values of Migrant-%-Population. The low and high values, respectively, are the mean values among countries in the bottom 25% and top 25% of Migrant-%-Population. Point estimates and confidence intervals of the simple slopes are also shown.

The simple slopes, all with negative point estimates, are significantly different from zero as none of the confidence intervals contain zero. The negative slopes for V-Ethnocultural (Panel A) and for V-Legitimation (Panel B) are both significantly steeper the lower the Migrant-%-Population, as suggested by the non-overlapping confidence intervals.

Thus, the more strongly citizens endorse V-Ethnocultural and V-Legitimation, the more negative their attitudes toward I-Cultural Impact; these associations are stronger in large migrant populations.

#### 6.4.3 Moderation by civil liberties

*H3*, particularly pertaining to the moderating effects of civil liberties, is partially supported. Tests of two-way interactions indicate that civil liberties moderate the effects of V-Legitimation, but not those of V-Ethnocultural or V-Civic (There is, however, a significant Migrant-%-Population X Civil Liberties X V-Ethnocultural, which is discussed in the next section.). Probing the significant moderation effect was done as in the preceding section.

**Figure 3:**
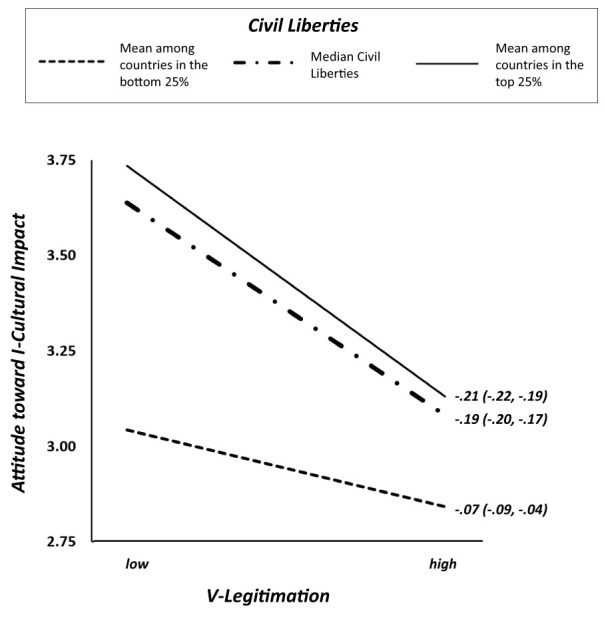
Countries' civil liberties moderate the effect of V-Legitimation on attitude toward I-Cultural Impact.

Figure 3 presents the interaction plot for V-Legitimation predicting attitude toward I-Cultural Impact at low, median, and high values of civil liberties. The simple slopes, all with negative point estimates, are significantly different from zero as none of the confidence intervals contain zero. The negative slopes at median and high values of civil liberties are steeper than at the low value of civil liberties, as suggested by the non-overlapping confidence intervals.

Thus, the more strongly citizens endorse V-Legitimation, the more negative their attitudes toward I-Cultural Impact; these associations are stronger under higher values of civil liberties.

#### 6.4.4 Migrant population size conditions the moderating effect of civil liberties

*H4* is partially supported. Migrant-%-Population conditions the moderation by civil liberties of the effect of V-Ethnocultural (i.e., the Migrant-%-Population X Civil Liberties X Ethnocultural interaction effect is significant). Probing this significant moderated moderation effect was done similarly to probing the significant moderation effects.

Although the Migrant-%-Population X Civil Liberties X Legitimation interaction effect is significant in Model 4, it is not robust, in various model specifications, to the removal of countries with poor factor and reliability statistics. Moreover, slope analyses and interaction plots for the Civil Liberties X Legitimation interaction effect, when compared across small, median, and large values of Migrant%-Population, show no differences that suggest a meaningful or substantive three-way interaction pattern.

**Figure 4:**
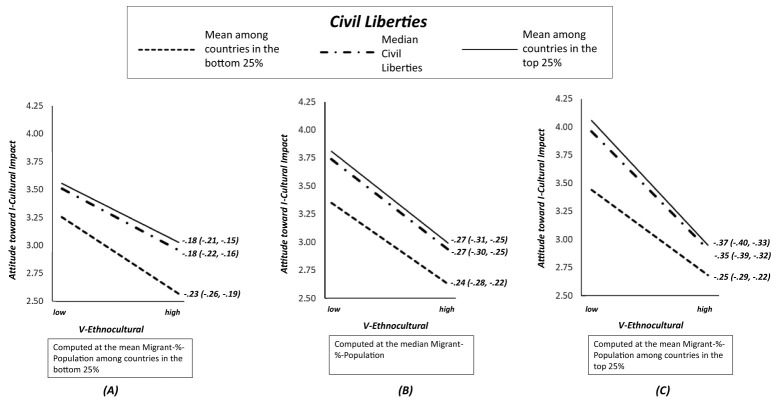
Civil liberties differentially moderate the negative ethnocultural effect on attitude toward I-Cultural Impact for countries with small, median, and large Migrant-%-Population.

Figure 4 presents the interaction plots for V-Ethnocultural predicting attitude toward I-Cultural Impact at low, median, and high values of civil liberties in the case of low, median, and high values of Migrant-%-Population.

At low Migrant-%-Population (Panel A), the negative slope is steeper at low than at median and high values of civil liberties. At median Migrant-%-Population (Panel B), there are no significant differences in the slopes of the three lines. At high Migrant-%-Population (Panel C), the negative slopes are steeper at median and high than at low values of civil liberties. Significant differences in slopes are indicated by the non-overlapping confidence intervals. These are verified by *t*-tests of difference between slopes, with the standard error of difference computed from the pertinent elements of the variance-covariance matrix of Model 4 (Raudenbush and Bryk, 2002).

Therefore, the moderating effect under the ethnocultural view shows that, with small migrant populations, civil liberties support positive views of immigrants' cultures: the stronger the civil liberties, the weaker the negative relationship between the ethnocultural view and attitudes toward I-Cultural Impact. With moderately sized migrant populations, civil liberties do not moderate this negative relationship. However, a tipping point emerges with large migrant populations, where civil liberties constrain positive views of immigrants' cultures: the stronger civil liberties, the stronger the negative relationship between the ethnocultural view and attitudes toward I-Cultural Impact.

## 7 Discussion

### 7.1 Strict boundary-setting through the ethnocultural and legitimation views

The results of this study show that citizens who believe that national identity is conferred on those who have the dominant ethnocultural heritage, or on those who have legitimated their claim to national identity, tend to hold less positive attitudes toward I-Cultural Impact, particularly in countries with relatively large migrant populations (i.e., significant moderation by migrant population size). On the other hand, a civic-oriented view of national identity tends to be associated with more positive attitudes across countries with varying migrant population sizes.

The results of this study suggest that the restrictive boundaries set by the ethnocultural and legitimation views may not support redefining the nation's cultural make-up to include the new or different cultures that immigrants bring. The reservation toward immigrant cultures posed by the ethnocultural criterion can be straightforwardly understood, as the basis of this criterion is cultural (belonging to the dominant ethnocultural heritage) and the object of evaluation of immigrants is also cultural (immigrants' cultural impact)

In contrast, legitimation criteria are not culture-based. Citizens' recognition of ingroup membership through legitimation—by virtue of citizenship, long-time residence, or birthright—does not directly invoke cultural lineage. Thus, the link between the legitimation view and negative attitudes toward I-Cultural Impact is not evident unless endorsers of the legitimation view also implicitly regard legitimated members as not descended from multigenerational citizens, particularly from the country's historical ethnocultural lineage. In this sense, although legitimation is the formal criterion for ingroup membership, legitimated members may still be seen as outside the original or core ingroup. This embedding of ethnocultural reasoning within the legitimation view is consistent with Kymlicka's (1995) supposition that citizens often expect immigrants to assimilate into the mainstream culture.

This study's results regarding the ethnocultural and legitimation views are similar not only in their main effects, but also in their moderation by civil liberties. Under stronger civil liberties, both views are more strongly associated with negative attitudes toward I-Cultural Impact. This pattern is discussed in Section 7.2. For the ethnocultural view, however, the pattern holds only under the large migrant populations, whereas for the legitimation view, this pattern holds regardless of migrant population size. How the moderation effect of civil liberties varies between the two views as a function of migrant population size is discussed in Section 7.3.

### 7.2 Distinct boundary-keeping mechanisms with the ethnocultural and legitimation views under strong civil liberties

This study finds that citizens in countries with stronger civil liberties hold more positive attitudes toward I-Cultural Impact, suggesting that civil liberties support cultural inclusion. Nonetheless, it also finds that strong civil liberties activate citizens' concerns about preserving the ingroup–outgroup boundary, thereby reinforcing their criteria for national identity and strengthening the association between these criteria and negative views about I-Cultural Impact.

This moderated relationship between citizens' ethnocultural view and their negative attitudes toward I-Cultural impact typifies results in the literature showing that frames or contexts ostensibly supportive of cultural diversity can instead prime citizens—particularly those with strong ethnic or national identification—to uphold the dominant culture and express prejudice toward immigrants' culture. Morrison et al.'s (2010) experiments show that highly identified white Americans, when primed with multiculturalism, exhibited higher social dominance and greater prejudice toward minorities, consistent with the assumption that they were more concerned than low-identified white Americans with maintaining the ingroup–outgroup boundary. Similar results are shown in Kauff et al. (2013), whose European survey data show that in countries with liberal integration policies, the negative relationship between right-wing authoritarianism and prodiversity beliefs is stronger. Their experiments showed that watching videos promoting multiculturalism or images of multicultural groups reduced prodiversity beliefs and increased prejudice toward minorities among participants high on right-wing authoritarianism.

Still another possible explanation for the stronger relationship between the ethnocultural view and negative attitudes toward I-Cultural Impact is that some citizens worry about a potential conflict between their country's strong civil liberties and immigrants' values and beliefs on issues such as gender equality, freedom of expression, and the influence of religion on government (Akkerman and Hagelund, 2007). Likewise, increasing racial diversity in the United States and globalization trends perceived as undermining American identity and dominance have been met with anxiety by higher-status groups (Mutz, 2018).

Thus, strong civil liberties uphold and protect outgroups' cultural identities and beliefs, enabling immigrants to influence or change the ingroup's cultural makeup. From a social identity perspective, this dynamic increases the permeability of the ingroup–outgroup boundary, creating tension for citizens who restrict the boundary with their ethnocultural view and thereby contributing to their negative attitudes toward I-Cultural impact.

This tension between increased boundary permeability and restricted boundary is likewise perceived by citizens with the legitimation view, albeit for a different underlying explanation. Under strong civil liberties, which acknowledge and protect citizens' freedoms and rights, the ingroup gains greater leverage over the outgroup, even as those freedoms and rights are extended, to some extent, to the outgroup. With the expanded liberties afforded to citizens, strong endorsers of the legitimation view are likely to assert their criteria by engaging in gatekeeping. When clear markers of citizenship, long-time residence, or birthright are unavailable, they may rely on alternative forms of gatekeeping, such as vigilance for evidence that immigrants are not assimilating to national norms.

Assimilation, however, implies expectations of conformity to cultural norms (Mouritsen, 2013). While the legitimation view is formally indifferent to immigrants' ethnocultural identity, increased boundary permeability may nevertheless foster a preference for monoculturalism, akin to the ethnocultural criteria. This overlap between legitimation-based gatekeeping and endorsement of cultural assimilation helps explain why multiculturalism sometimes elicits positive reactions and at other times negative ones (Morrison et al., 2010). Overall, the results provide evidence that the ethnocultural and legitimation views enforce stricter boundary-keeping when there is increased boundary permeability under strong civil liberties.

### 7.3 Distinct boundary-keeping mechanisms with the ethnocultural and legitimation views across different migrant population sizes

This study finds a moderation by migrant population size with both the ethnocultural and legitimation views: the larger the migrant population size, the stronger the association between these views and negative attitudes toward I-Cultural Impact. A large migrant population may make ethnocultural diversity more salient, or heighten concerns about the legitimate status of migrants, thereby increasing the perceived threat to ingroup membership among endorsers of these views. From the lens of social identity theory, a large migrant population reinforces citizens' restrictive ingroup–outgroup boundary-setting.

A consideration of migrant population size shows that civil liberties differentially moderate the associations of the ethnocultural and legitimation views with attitudes toward I-Cultural Impact. With the ethnocultural view, civil liberties weaken its association with negative attitudes toward I-Cultural impact but, notably, only in countries with small migrant populations. This pattern reverses as migrant populations increase. With moderately sized migrant populations, civil liberties do not moderate this negative association. With large migrant populations, civil liberties instead strengthen the association between the ethnocultural view and negative attitudes toward I-Cultural Impact, suggesting a tipping point at which the permeability of the ingroup–outgroup boundary under strong civil liberties is outweighed by citizens' restrictive ingroup–outgroup boundary under high migrant density.

This tension between civil liberties—which increase boundary permeability—and exclusionary criteria for national identity—which restrict the boundary—is more widespread with the legitimation view. Across small, medium, and large migrant population sizes, civil liberties consistently strengthen the association between the legitimation view and negative attitudes toward I-Cultural Impact.

These differential effects of migrant population size can be explained by how national membership criteria dictate boundary-keeping mechanisms. In brief, ethnocultural criteria prompt endorsers to identify outgroup members by their ethnocultural identities; legitimation criteria prompt endorsers to assess outgroup members' qualifications for legitimated membership. While dominant ethnocultural identity often has visible markers, for example, in persons' appearance, behaviors, and speech, legitimated national identity cannot be readily inferred from these or similar markers.

Because legitimation criteria are difficult to verify directly, increased boundary permeability under strong civil liberties creates uncertainty about who is a legitimate ingroup member. Since this uncertainty exists regardless of migrant population size, endorsers may engage in gatekeeping as a matter of course, or even persistently, and may rely on indirect indicators of legitimation, such as assimilation or adherence to norms.

Thus, from a social identity theory perspective, a large migrant population signals increased permeability of the boundary set by endorsers of the ethnocultural view. It does not signal, to the same extent, increased permeability of the boundary set by endorsers of the legitimation view. As a result, the tension between boundary permeability and boundary restriction surfaces under a large migrant population among endorsers of the ethnocultural view, whereas this tension remains among endorsers of the legitimation view regardless of migrant population size.

Although legitimation is not identical to assimilation, the logic of boundary-keeping may be similar. Empirical findings that assimilation is widely preferred across levels of diversity in society (Verkuyten, 2005) or across various contexts (Callens et al., 2019) lend plausibility to the idea that expectations of and vigilance toward legitimation persist across migrant population sizes.

### 7.4 The civic view vs. the ethnocultural view: varying openness to cultural inclusion

This study finds a positive relationship between the civic view and positive attitudes toward I-Cultural Impact. This relationship is robust, with no evidence of moderation by civil liberties or migrant population size. From the lens of social identity theory, this finding suggests that the civic view's expansive boundary-setting facilitates outgroup members' inclusion in the ingroup regardless of variations in boundary permeability associated with variations in civil liberties and migrant population size.

Compared to the ethnocultural and legitimation views, the civic view facilitates the inclusion of immigrant cultures in a country's cultural make-up. A civic identity can be shared by a larger group of people, abating antipathy between citizens and immigrants (Miller and Sundas, 2014). More importantly, for immigrants, the civic view provides a notion of national identity that is not only a matter of legitimated membership or ethnocultural origins, but also a deep, personal sense of belonging to a nation (Georgiadis and Manning, 2013).

The civic criteria for national membership, which are respect for and a feeling of belonging to a nation, provide what Falomir-Pichastor and Mugny (2013) refer to as a higher-level categorization of peoples that supersedes the separation between ingroup and outgroup. A proposition of social identity theory is that people often define their identities in terms of the group to which they feel attachment, belonging, and a sense of obligation (Tajfel and Turner, 1986). From a civic perspective, as the nation is built on its institutions and laws, so is national identity defined by respect for them. The civic view may thus hold the key to a strong national identity among a diverse populace.

Although many countries' definitions of national identification have shifted to a civic model (Jones and Smith, 2001), a cultural construction of national identity has been shown to lead to a stronger attachment to the nation than does a civic construction (Miller and Sundas, 2014). In other words, the civic view may offer an inclusive but weak national identity, whereas the ethnocultural view may offer a strong but exclusive one (Miller and Sundas, 2014). The promise, then, of the civic view needs to be examined together with its precariousness.

### 7.5 Limitations and future research

We note the following limitations of this study in its measures and scope. First, the indicators of civil liberties used in this study cover a broad range rather than being specific to immigrants or minority groups. Likewise, attitudes toward I-Cultural impact can be measured with larger, more nuanced scales. Administering an international survey that covers various constructs, such as the ISSP, however, constrains the number of items per construct.

Second, the quantitative approach of this study, together with the *a priori*-specified country-level dimensions, does not capture broader cross-national heterogeneity that shapes citizens' views on national identity and immigrant cultures. Such heterogeneity can be better examined through qualitative analyses and comparative country reports that situate issues within countries' sociocultural, historical, and political contexts. Beyond developing country scenarios that reflect the results of this study, comparative country reports can address issues, such as the sociodemographic and ethnocultural profiles of immigrants relative to the established population; the fairness with which states are perceived to treat immigrants' freedoms and rights compared to the established population; the country's immigrant profiles vis á vis the citizens' economic, political, and cultural preparedness to receive immigrants; and the implications of countries' cultural integration policies for citizens' attitudes toward cultural diversity and inclusion.

For related future research, it is worthwhile to examine other possible context moderators of the relationship between citizens' criteria for national membership and their attitudes toward immigrants' cultures. (Bloemraad and Schönwälder 2013) note features that suggest integration at the same time that they suggest non-integration, or signs of citizenship that are now being regarded as signs of dis-citizenship. Miller and Sundas (2014) share the same perspective: whether a factor will support national identity (e.g., providing a source of social unity) or undermine it (e.g., questioning the social justice of a welfare state) is highly dependent on the country context. Relatedly, the target immigrant group can serve as a moderator of the influence of national identity views on attitudes toward I-Cultural Impact (Nagayoshi, 2011). For instance, people in Germany may be more receptive to the culture of immigrants from Austria, but less so to that of immigrants from Syria (Dustmann and Preston, 2007). Likewise, people in the United Kingdom may be more receptive to the culture brought by immigrants from Australia, but less so to that from Pakistan (Ford, 2011).

### 7.6 The contextual complexity of cultural inclusion

In sum, the results of this study show that the ingroup–outgroup boundary and its permeability depend, respectively, on citizens' national identity criteria and the country contexts of civil liberties and migrant population size. Processes through which individuals come to acknowledge the “other” as having the same identity as themselves (Herrmann and Brewer, 2004) potentially move the boundary to include more immigrants to the ingroup. Pehrson et al. (2009) contest a fixed ethnic-civic distinction and instead recommend examining national identity criteria from the ground up, particularly, within what they call “argumentative contexts.” In general, therefore, as regards empirical research and policy studies, it is advantageous to assume that boundaries are shifting and their permeability is flexible.

In this 34-country study, we situate the discourse on migration and citizenship within intercultural spaces shared by citizens, who are recognized members of the nation, and migrants, who must negotiate their place within it. We examine how citizens' inclusionary and exclusionary constructions of national identity, along with countries' migrant demographics and civil liberties, shape citizens' readiness for the cultural diversity associated with international migration. Within the study's delimitation of social, cultural, and demographic contexts to civil liberties and migrant population size, the findings reveal the complexity of the mechanisms and conditions operating at the migration-citizenship nexus. These results can be understood through the lens of the superdiversity framework, which construes diversity as emerging from interlocking demographic, social, and cultural dynamics (Meissner and Vertovec, 2015; Vertovec, 2007, 2019). From this perspective, the fluidity of national identity, alongside the complex social, cultural, and demographic phenomena surrounding human mobility (Vertovec, 2023), constitutes a fulcrum on which a country's multicultural inclusion stands.

## Data Availability

Publicly available datasets were analyzed in this study. This data can be found here: The individual-level data were obtained from the 2013 National Identity III dataset, which is on public domain: https://www.gesis.org/en/issp/data-and-documentation/national-identity/2013. The country-level statistics are also on public domain: https://www.immigration.gov.tw/5475/5478/141478/141380/141776/cp_news; https://www.nationmaster.com/country-info/profiles/Taiwan/People; https://siyosat.wordpress.com/wp-content/uploads/2014/10/democracy_index_2013_web-2.pdf; https://www.un-ilibrary.org/content/books/9789210564267.
