# Supplementary material for: Citizens' national identity criteria and attitudes toward immigrants' cultural impact: ingroup–outgroup boundary setting and permeability across national contexts
Source: Front Sociol. 2026 Jul 20;11:1849822. doi: 10.3389/fsoc.2026.1849822 (PMC13430557; doi:10.3389/fsoc.2026.1849822)
Supplement: Supplementary file 1 [file Supplementary_file_1.pdf]

Section 1

Respondents' Demographic Profiles per Country

Country	Sample size	Age M (SD)	% female	% with post-secondary education	% living in urban areas	% with both parents citizens
Belgium	1873	51.1 (17.9)	53	43	33	87
Croatia	993	44.4 (16.3)	51	22	45	92
Czech Republic	1883	46.6 (16.8)	51	14	32	95
Denmark	1277	46.9 (16.9)	49	84	40	95
Estonia	869	47.4 (17.4)	63	52	45	77
Finland	1203	47.0 (16.9)	54	49	44	98
France	1908	54.1 (17.7)	49	39	31	92
Georgia	1490	48.3 (17.8)	63	64	35	98
Germany	1613	49.7 (17.7)	50	86	30	92
Great Britain	784	52.1 (18.3)	57	36	32	90
Hungary	1001	48.6 (17.1)	56	19	40	96
Iceland	1043	45.5 (18.4)	49	47	65	95
India	1526	40.9 (14.3)	41	11	23	95
Ireland	1095	52.0 (14.5)	64	58	37	94
Israel	1179	47.4 (19.2)	48	48	60	63
Japan	1207	51.6 (18.5)	53	35	31	99
Latvia	867	43.2 (15.5)	59	63	45	78
Lithuania	1182	48.2 (17.8)	59	54	38	95
Mexico	1010	41.1 (16.0)	51	55	61	96
Netherlands	1513	56.0 (16.0)	47	40	90	95
Norway	1428	49.0 (16.2)	52	55	39	93
Philippines	1193	43.2 (16.1)	50	31	44	99
Portugal	974	49.5 (18.0)	55	16	46	96
Russia	1511	48.4 (18.3)	64	31	48	97
Slovak Republic	1155	48.2 (16.3)	58	17	20	95
Slovenia	989	50.4 (18.0)	56	30	20	95
South Africa	2547	41.9 (17.6)	61	11	73	94
South Korea	1283	44.3 (16.6)	50	54	57	99
Spain	1125	49.1 (17.5)	52	16	32	96
Sweden	1024	51.3 (16.6)	52	43	45	87
Switzerland	1018	50.1 (17.9)	51	77	16	78
Taiwan	1952	46.0 (16.9)	50	43	57	97
Turkey	1653	40.8 (14.9)	49	13	56	97
United States	1186	49.7 (17.6)	54	40	85	88
