# Supplementary material for: Citizens' national identity criteria and attitudes toward immigrants' cultural impact: ingroup–outgroup boundary setting and permeability across national contexts
Source: Front Sociol. 2026 Jul 20;11:1849822. doi: 10.3389/fsoc.2026.1849822 (PMC13430557; doi:10.3389/fsoc.2026.1849822)
Supplement: Supplementary file 2 [file Supplementary_file_2.pdf]

Section 2
Factor and Item Reliability Analyses of Individual-Level Variables per Country

Table 1
Factor and Item Reliability Analyses for V-Ethnocultural per Country

Country	Proportion of Total Variance Explained by a One-Factor Solution	Average Item Loading on the Factor	Internal Consistency (Cronbach's α)	Average Interitem Correlation
Belgium	.50	.67	.48	.23
Croatia	.64	.80	.72	.46
Czech Republic	.47	.68	.42	.20
Denmark	.61	.78	.68	.42
Estonia	.46	.67	.41	.18
Finland	.60	.77	.67	.40
France	.56	.74	.59	.33
Georgia	.58	.76	.63	.37
Germany	.50	.70	.49	.24
Great Britain	.56	.75	.62	.36
Hungary	.48	.69	.44	.22
Iceland	.59	.77	.65	.39
India	.54	.73	.57	.31
Ireland	.59	.77	.66	.39
Israel	.57	.75	.62	.35
Japan	.63	.79	.71	.45
Latvia	.50	.70	.50	.25
Lithuania	.53	.72	.53	.29
Mexico	.64	.80	.71	.47
Netherlands	.51	.68	.54	.25
Norway	.57	.75	.61	.35
Philippines	.58	.76	.59	.37
Portugal	.49	.69	.46	.22
Russia	.62	.79	.68	.43
Slovakia	.51	.71	.51	.27
Slovenia	.55	.73	.59	.32
South Africa	.54	.74	.57	.31
South Korea	.62	.79	.69	.43
Spain	.57	.76	.62	.36
Sweden	.55	.74	.59	.33
Switzerland	.53	.70	.56	.28
Taiwan	.54	.83	.56	.30
Turkey	.69	.83	.77	.54
United States	.65	.80	.72	.48

Table 2
Factor and Item Reliability Analyses for V-Legitimation per Country

Country	Proportion of Total Variance Explained by a One-Factor Solution	Average Item Loading on the Factor	Internal Consistency (Cronbach's α)	Average Interitem Correlation
Belgium	.69	.83	.76	.53
Croatia	.71	.86	.80	.57
Czech Republic	.68	.82	.76	.52
Denmark	.70	.84	.79	.56
Estonia	.67	.82	.75	.50
Finland	.74	.86	.83	.62
France	.64	.80	.71	.46
Georgia	.68	.82	.75	.51
Germany	.71	.84	.80	.57
Great Britain	.70	.84	.78	.55
Hungary	.71	.85	.82	.61
Iceland	.66	.81	.73	.49
India	.50	.71	.48	.25
Ireland	.72	.85	.80	.57
Israel	.56	.74	.58	.34
Japan	.66	.81	.74	.49
Latvia	.69	.83	.78	.54
Lithuania	.68	.82	.76	.52
Mexico	.79	.89	.86	.68
Netherlands	.67	.82	.76	.51
Norway	.67	.82	.75	.51
Philippines	.59	.77	.65	.39
Portugal	.65	.80	.72	.47
Russia	.67	.82	.75	.50
Slovakia	.69	.83	.78	.54
Slovenia	.72	.85	.80	.58
South Africa	.65	.80	.72	.47
South Korea	.71	.84	.79	.56
Spain	.75	.86	.83	.62
Sweden	.68	.83	.77	.53
Switzerland	.67	.82	.76	.51
Taiwan	.66	.81	.74	.49
Turkey	.79	.89	.87	.69
United States	.67	.82	.75	.52

Table 3

Factor and Item Reliability Analyses for V-Civic per Country

Country	Proportion of Total Variance Explained by a One-Factor Solution	Average Item Loading on the Factor	Internal Consistency (Cronbach's α)	Average Interitem Correlation
Belgium	.68	.82	.52	.35
Croatia	.69	.83	.54	.37
Czech Republic	.69	.83	.56	.39
Denmark	.62	.79	.37	.24
Estonia	.66	.82	.48	.33
Finland	.63	.79	.41	.26
France	.67	.82	.49	.35
Georgia	.67	.82	.50	.34
Germany	.67	.82	.49	.33
Great Britain	.69	.83	.54	.38
Hungary	.63	.80	.41	.27
Iceland	.62	.79	.38	.24
India	.69	.83	.54	.37
Ireland	.65	.80	.45	.29
Israel	.72	.85	.61	.43
Japan	.69	.83	.55	.38
Latvia	.78	.88	.72	.56
Lithuania	.74	.86	.65	.48
Mexico	.71	.84	.59	.42
Netherlands	.64	.80	.43	.27
Norway	.56	.75	.21	.13
Philippines	.70	.83	.53	.38
Portugal	.69	.83	.54	.37
Russia	.74	.86	.65	.48
Slovakia	.72	.85	.60	.44
Slovenia	.70	.84	.57	.40
South Africa	.69	.83	.53	.38
South Korea	.70	.84	.60	.40
Spain	.70	.84	.58	.41
Sweden	.60	.78	.31	.20
Switzerland	.63	.79	.39	.25
Taiwan	.76	.87	.69	.53
Turkey	.71	.84	.59	.43
United States	.63	.79	.41	.26

Table 4
Factor and Item Reliability Analyses for Attitude toward I-Cultural Impact per Country

Country	Proportion of Total Variance Explained by a One-Factor Solution	Average Item Loading on the Factor	Internal Consistency (Cronbach's α)	Average Interitem Correlation
Belgium	.77	.88	.69	.53
Croatia	.63	.80	.42	.27
Czech Republic	.65	.81	.46	.30
Denmark	.75	.86	.66	.50
Estonia	.67	.82	.50	.33
Finland	.81	.90	.76	.61
France	.78	.88	.71	.56
Georgia	.69	.83	.55	.38
Germany	.74	.86	.64	.48
Great Britain	.77	.88	.70	.54
Hungary	.67	.82	.50	.34
Iceland	.72	.85	.61	.44
India	.67	.00[a]	-1.02[a]	-.33[a]
Ireland	.73	.85	.63	.46
Israel	.63	.79	.42	.26
Japan	.60	.78	.33	.20
Latvia	.70	.83	.56	.39
Lithuania	.69	.83	.54	.37
Mexico	.51	.00[a]	-.04[a]	-.02[a]
Netherlands	.73	.86	.63	.47
Norway	.80	.89	.74	.59
Philippines	.51	.71	.02[a]	.01[a]
Portugal	.73	.86	.63	.47
Russia	.59	.77	.31	.19
Slovakia	.65	.81	.46	.30
Slovenia	.69	.83	.54	.37
South Africa	.53	.73	.11	.06
South Korea	.61	.78	.35	.22
Spain	.65	.81	.46	.30
Sweden	.79	.89	.73	.59
Switzerland	.71	.85	.59	.43
Taiwan	.65	.81	.47	.31
Turkey	.50	.71	.01[a]	.00[a]
United States	.85	.72	.60	.43

[a] These are outlying values indicating that responses to the items *Immigrants improve [country's nationality] society by bringing new ideas and cultures; and [Country's] culture is generally undermined by immigrants* (reversed-scoring) have zero or near-zero correlation (for Philippines and Turkey), or are negatively correlated (for India and Mexico).
Note. Multilevel modeling without the India and Mexico data yielded the same results as those reported in the paper.
