# Supplementary material for: Citizens' national identity criteria and attitudes toward immigrants' cultural impact: ingroup–outgroup boundary setting and permeability across national contexts
Source: Front Sociol. 2026 Jul 20;11:1849822. doi: 10.3389/fsoc.2026.1849822 (PMC13430557; doi:10.3389/fsoc.2026.1849822)
Supplement: Supplementary file 3 [file Supplementary_file_3.pdf]

Section 3

Imputation Method for Missing Data

Missing data on the items for V-Ethnocultural, V-Legitimation, V-Civic, and attitude toward I-Cultural Impact averaged 2.37%, 1.68%, 2.16%, and 5.01%, respectively. If imputation was not done and participants with at least one missing datum on these items were excluded, only 87% of the records (38,758 of 44,554) would remain.

Missing data were imputed by training a neural network using the backpropagation algorithm and a categorical loss function in the Python deep learning library Keras (Keras Special Interest Group, n.d.). The input data comprised items on national identity views, attitude toward I-Cultural Impact, and country membership. To train the network to predict a missing value for an item, data from respondents with non-missing values on this item were randomly split into training (n = 35,000) and test data. The network was trained to predict a response to an item using the training data. Training iterations (epochs) were terminated when training data prediction accuracy continued to improve without a corresponding increase in test data accuracy (i.e., overfitting).

Closed formula imputation methods based on known probability distributions (e.g., Gelman & Hill, 2006; Plumpton et al., 2016) do not use test data to compare imputed and actual values and thus do not arrive at prediction accuracy. The neural network algorithm used here does.

The neural network correctly predicted, on average, 63.7%, 67.6%, 67.5%, and 45.9% of the test data for V-Ethnocultural, V-Legitimation, V-Civic, and attitude toward I-Cultural Impact, respectively. Albeit prediction accuracies were low, the correlations between actual and predicted values for the test data were large, averaging .63, .64, .51, and .43, respectively, for V-Ethnocultural, V-Legitimation, V-Civic, and attitude toward I-Cultural Impact. Furthermore, for the V-Ethnocultural, V-Legitimation, and V-Civic items (on a 4-point scale), 93.8%, 97.0%, and 95.7%, respectively, of the inaccurately predicted values differed from the actual values by .5 or 1 point. For the attitude toward I-Cultural Impact items (on a 5-point scale), 92.8% of the inaccurately predicted values differed from the actual values by 1 or 2 points.

Reference:

Gelman, A., and Hill, J. (2006). *Data Analysis Using Regression and Multilevel/Hierarchical Models.* Cambridge University Press.

Keras Special Interest Group. (n.d.). *Keras: The Python deep learning library.* [Computer software]. Available online at: https://keras.io/ [Accessed March 19, 2026].

Plumpton, C. O., Morris, T., Hughes, D. A., and White, I. R. (2016). Multiple imputation of multiple multi-item scales when a full imputation model is infeasible. *BMC Res. Notes*. 9, 1-15. doi:10.1186/s13104-016-1853-5
