# Supplementary material for: Citizens' national identity criteria and attitudes toward immigrants' cultural impact: ingroup–outgroup boundary setting and permeability across national contexts
Source: Front Sociol. 2026 Jul 20;11:1849822. doi: 10.3389/fsoc.2026.1849822 (PMC13430557; doi:10.3389/fsoc.2026.1849822)
Supplement: Supplementary file 4 [file Supplementary_file_4.pdf]

Section 4

The Nested Multilevel Models Tested

Model 0<br>Baseline,<br>fully unconditional model	Model 1<br>Level-1 predictors<br>and covariates added	Model 2<br>Level-2 predictors added	Model 3<br>Two-way cross-level<br>interactions added	Model 4<br>Three-way cross-level<br>interactions added
**Level-1 model for I-Cultures receptivity**				
Intercept β_{0j} [a]<br>+ level-1 residual r_{ij} [b]	$\beta_{0j} + \Sigma\, \beta x + r_{ij}$ [c]<br>β=regression coefficient/slope<br>x=predictor/covariate	$\beta_{0j} + \Sigma\, \beta x + r_{ij}$	$\beta_{0j} + \Sigma\, \beta x + r_{ij}$	$\beta_{0j} + \Sigma\, \beta x + r_{ij}$
**Level-2 model for the level-1 intercept β_{0j}**				
Fixed intercept γ_{00} [d]<br>+ level-2 residual u_{0j}[a]	$\gamma_{00} + u_{0j}$	γ_{00} +<br>γ_{01} *migrant-% +<br>γ_{02} *civil liberties + u_{0j}	γ_{00} +<br>γ_{01} *migrant-% +<br>γ_{02} *civil liberties + u_{0j}	γ_{00} +<br>γ_{01} *migrant-% +<br>γ_{02} *civil liberties + u_{0j}
**Level-2 model for the βs of level-1 covariates**				
There are no covariates.	Fixed intercept γ<br>There is no random component/level-2 residual u.	γ [e]	γ	γ
**Level-2 model for the βs of level-1 predictors**				
There are no predictors.	Fixed intercept γ<br>There is no random component/level-2 residual u	γ	γ +<br>γ_1*migrant-% +<br>γ_2*civil liberties[f]	γ +<br>γ_1 *migrant-% +<br>γ_2*civil liberties +<br>γ_3 *migrant-%*civil liberties[g]

[a] varies across countries. Subscript j refers to countries.

[b] varies across individuals within countries. Subscripts i and j refer to individuals and countries, respectively.

[c] Subscripts for β and x are omitted, but there are different estimates of β for the different national identity views x. $\Sigma\, \beta x$ is the sum of the product between β and x.

[d] does not vary across countries.

[e] Subscripts for γ are omitted, but there are different estimates of γ for the different covariates and predictors.

[f] Model 3: At level-2, $\beta = \gamma + \gamma_1$ *migrant-% + γ_2*civil liberties. Substituting the right-hand term to βx in the level-1 equation will generate the terms γ_1 *x*migrant-% and γ_2* x*civil liberties (where x is a national identity view). x*migrant-% is the two-way interaction between a national identity view and migrant-%. x*civil liberties is the two-way interaction between a national identity view and civil liberties.

[g] Model 4: At level-2, $\beta = \gamma + \gamma_1$*migrant-% + γ_2*civil liberties + γ_3 *migrant-%*civil liberties. Substituting the right-hand term to βx in the level-1 equation will generate the term γ_3 * x *migrant-%*civil liberties (where x is a national identity view). x *migrant-%*civil liberties are three-way interactions among a national identity view, migrant-%, and civil liberties.
